# Acquired immune deficiency syndrome-related intravascular large B-cell lymphoma primarily arising from lymph nodes: a case report

**DOI:** 10.3389/fonc.2026.1767472

**Published:** 2026-01-30

**Authors:** Wei Zhang, Qi sui Li, Chang Gang Deng, Jing Yuan

**Affiliations:** Infection Department of Chongqing Public Health Medical Center, Chongqing, China

**Keywords:** acquired immune deficiency syndrome, case report, HIV, intravascular large B-cell lymphoma, lymph nodes

## Abstract

**Background:**

Intravascular large B-cell lymphoma (IVLBCL) is a rare subtype of diffuse large B-cell lymphoma, with nodal involvement being particularly uncommon. Due to its atypical clinical presentation, timely and accurate diagnosis is often challenging. Positron emission tomography–computed tomography (PET-CT) and pathological biopsy can assist in the diagnostic process.

**Case description:**

A 52-year-old woman with acquired immune deficiency syndrome (AIDS) and a one-year history of chronic hepatitis B was admitted to the Infection Department of Chongqing Public Medical Center in September 2021. She had been on long-term antiviral therapy with Lamivudine, Tenofovir, and Efavirenz (3TC/TDF/EFV). Her chief complaints included a left groin mass, fever, and significant weight loss. Surgical excision and pathological examination of the left inguinal lymph nodes confirmed the diagnosis of IVLBCL. Following effective antiretroviral therapy and six cycles of CHOP/R-CHOP chemotherapy, the patient achieved complete remission. The patient remained free of lymphoma recurrence during the two-year follow-up; passed away due to COVID-19 in March 2023.

**Conclusion:**

This case illustrates four critical teaching points: (1) intravascular large B-cell lymphoma (IVLBCL) is a rare subtype of DLBCL; (2) isolated lymph node involvement is exceptionally uncommon in reported cases; (3) the absence of typical cutaneous or central nervous system involvement further complicates the diagnosis; and (4) its occurrence in an HIV-positive patient represents a particularly unusual clinical scenario. Early recognition of these atypical features, together with prompt combined antiretroviral and chemotherapy, achieved complete remission despite profound immunosuppression, highlighting the need for vigilant and individualized management in such rare and diagnostically challenging presentations.

## Introduction

Intravascular large B-cell lymphoma (IVLBCL), also referred to as angiotropic large cell lymphoma, is a rare extranodal subtype of diffuse large B-cell lymphoma (DLBCL). It predominantly affects elderly individuals, typically between 60 and 80 years of age, and is characterized by the selective proliferation of neoplastic cells within the lumina of small blood vessels, particularly capillaries (1). Two major clinical variants have been identified: the Western variant, which often presents with cutaneous and neurological manifestations, and the Asian variant, frequently associated with hemophagocytic syndrome. Although IVLBCL commonly involves extranodal sites and directly infiltrates multiple organs, lymph node involvement is rare (2). This report describes an unusual case of primary nodal IVLBCL in an HIV-positive patient from Chongqing Public Health Medical Center, who attained complete remission following antiretroviral therapy and six cycles of chemotherapy. The patient remained free of lymphoma recurrence during the two-year follow-up; passed away due to COVID-19 in March 2023.

## Case presentation

A 52-year-old Chinese female from Sichuan Province was diagnosed with HIV infection one year prior to admission and began combined antiretroviral therapy (cART) with 3TC/TDF/EFV. She was admitted to the Department of Infectious Diseases on September 20, 2021, due to recent onset of a left groin mass, fever (37.8°C), and a 5 kg weight loss. The patient reported discontinuation of antiretroviral treatment for two months before admission. Physical examination revealed a left groin mass without skin lesions or neurological abnormalities.

Laboratory findings at admission included: CD4+ count 32 cells/μL, CD4/CD8 ratio 0.07, HIV viral load <50 copies/mL; hemoglobin 79 g/L, MCV 84.40 fL; leukocyte count 5.01×10^9^/L with lymphopenia (0.68×10^9^/L); platelet count 315×10^9^/L; CRP 143.49 mg/L; albumin 28.8 g/L; LDH 860 U/L; normal liver and renal function. Viral hepatitis serology was positive for HBsAg, HBeAb, and HBcAb, with undetectable HBV DNA; EBV-DNA was positive. Tuberculosis DNA was detected, while tests for cytomegalovirus, herpes simplex virus, and other infections were negative. Bone marrow examination and cerebrospinal fluid analysis showed no abnormalities.

**Figure 1:**
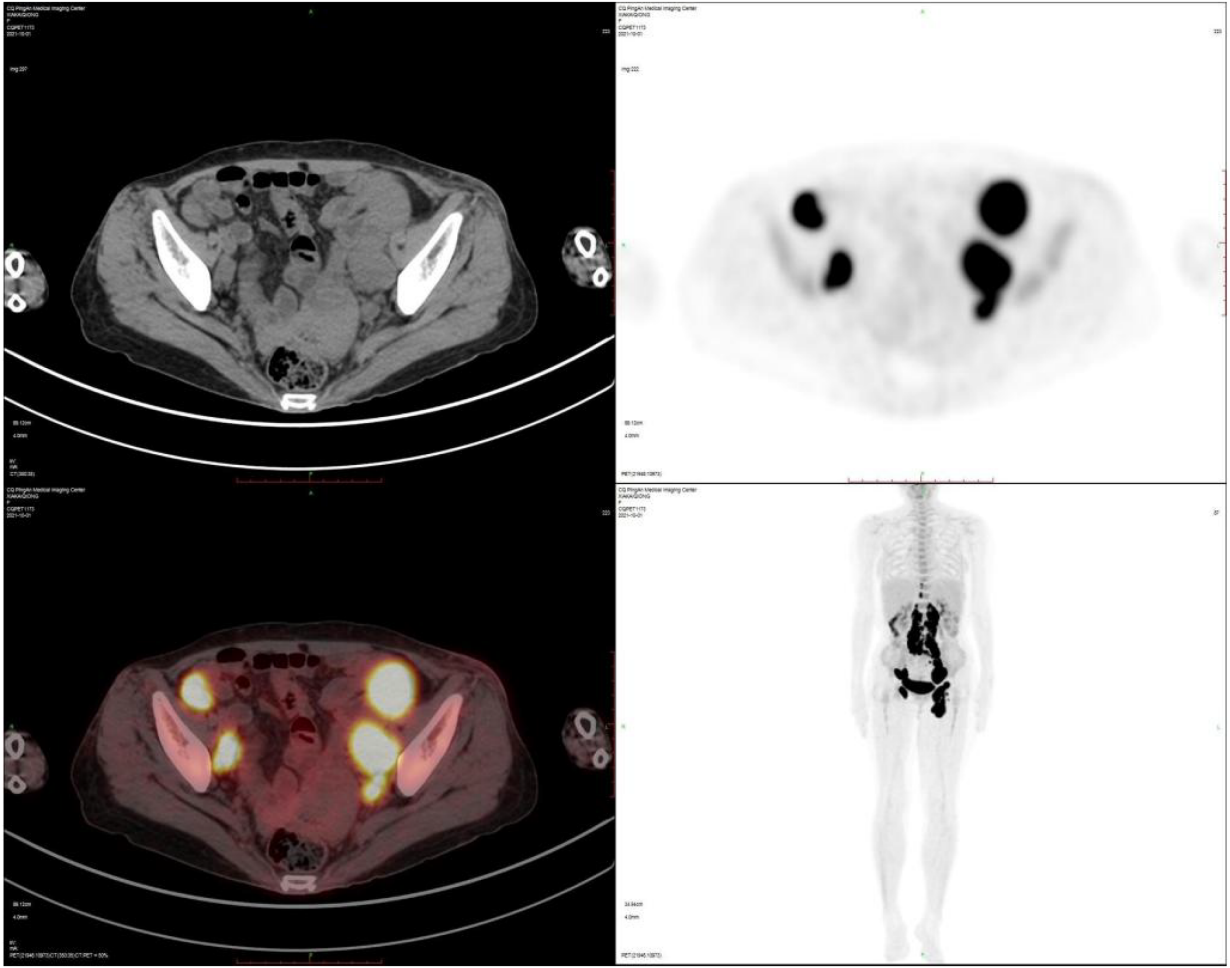
PET-CT before treatment: The scan reveals hypermetabolic lymph nodes (Deauville score 5) involving the bilateral inguinal, retrocrural, retroperitoneal, and pelvic regions.

**Figure 2:**
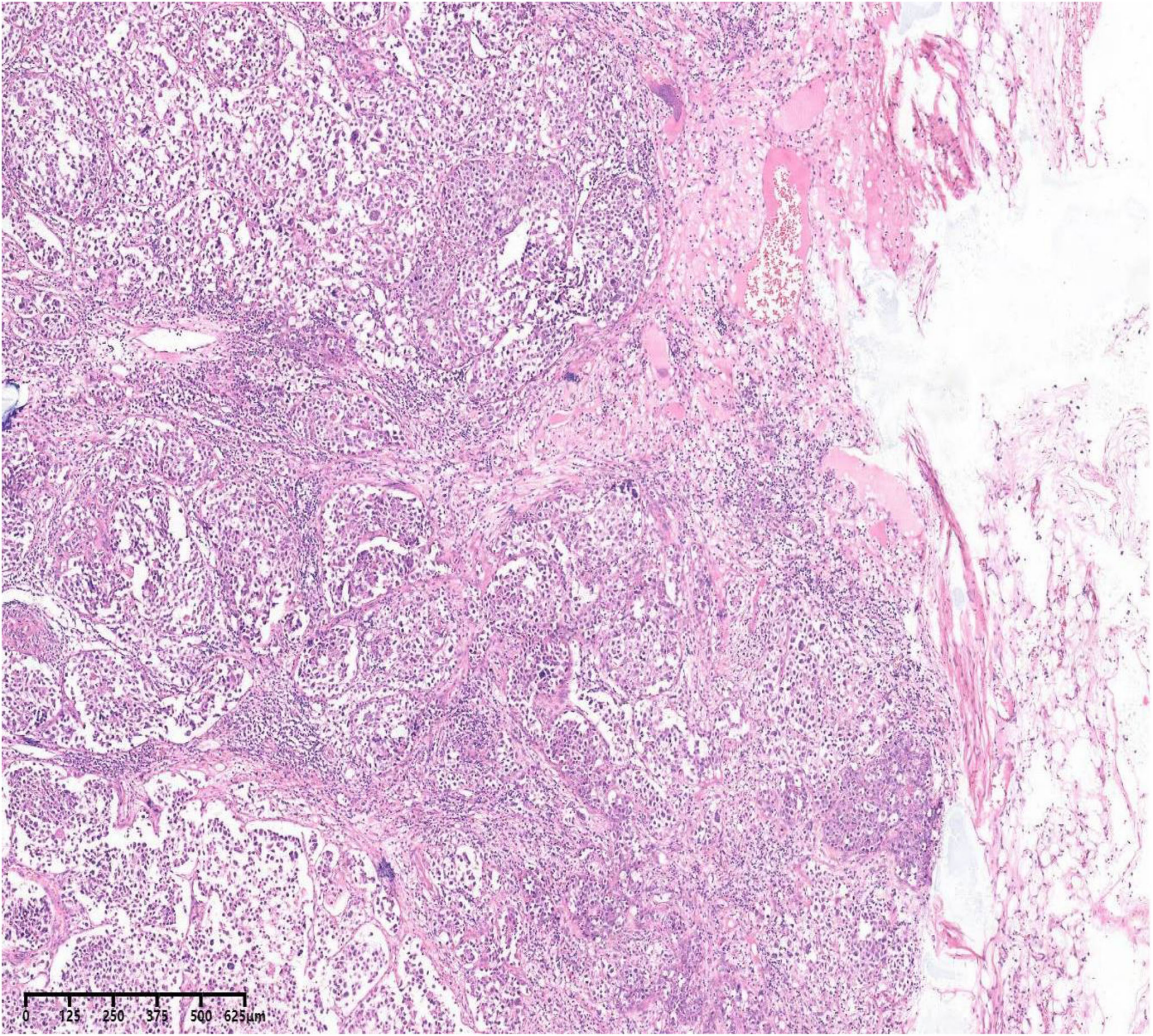
H&E stain,40. The lymph node capsule was visible Abnormal intravascular growth of tumor cells, with abundant cytoplasm, large nuclei, obvious nucleoli, and common mitosis.

**Figure 3:**
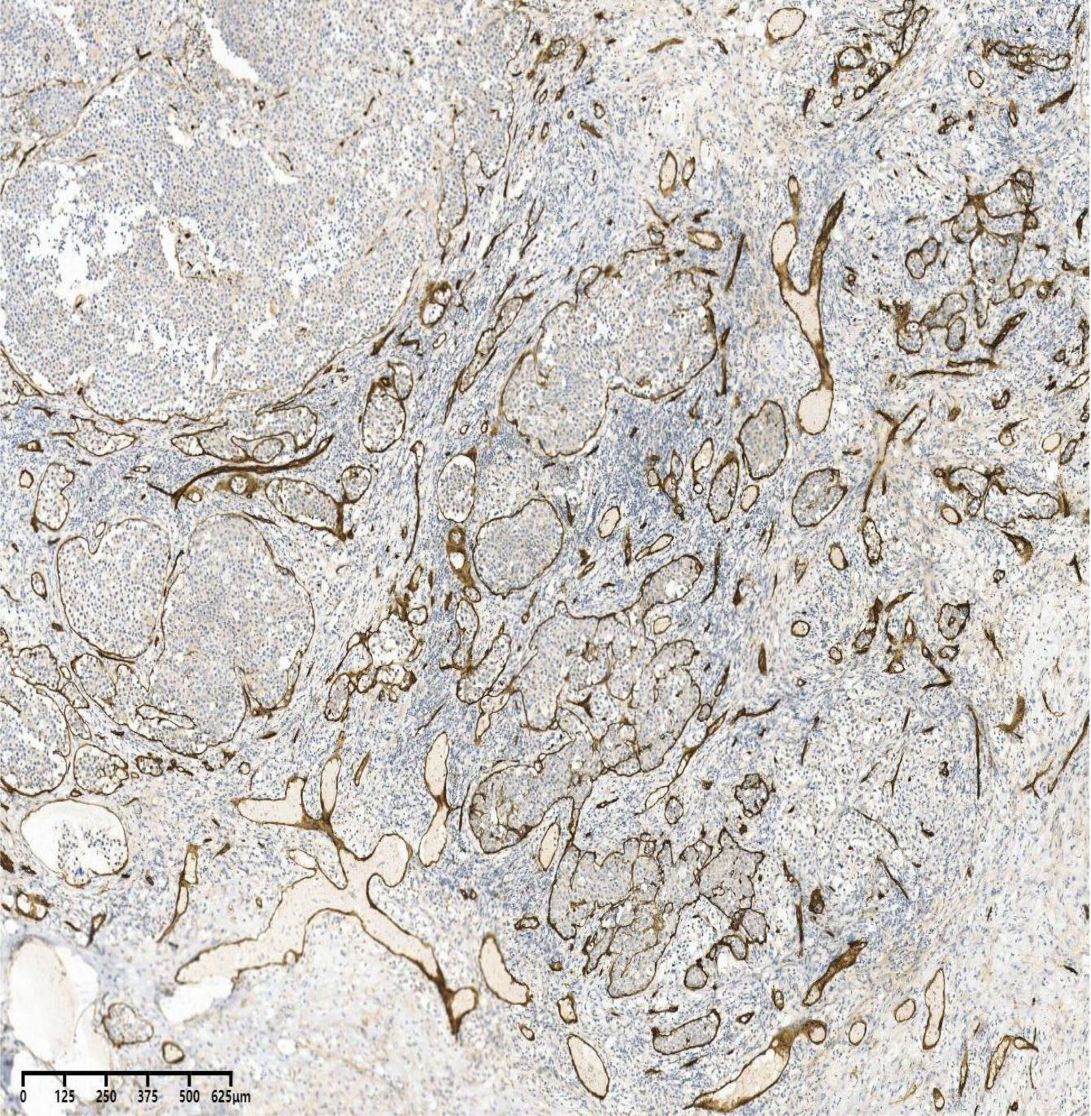
CD34 +,40×. CD34 positivity clearly demonstrates tumor cells within the vascular lumen.

Imaging studies demonstrated an enlarged left inguinal lymph node (49×23×36 mm) with vascular signals on ultrasound. PET-CT revealed hypermetabolism (Deauville score5) in bilateral inguinal, retrocrural, retroperitoneal, and pelvic lymph nodes (**Figure 1**). Left inguinal lymph node biopsy confirmed intravascular large B-cell lymphoma, showing tumor cells with abundant cytoplasm, large nuclei, prominent nucleoli, and frequent mitoses within vascular spaces (**Figures 2**, **3**).

Immunohistochemical analysis showed positivity for CD20, CD30,CD31,CD34, PAX-5, Bcl-2, Bcl-6, MUM-1, c-MYC, P16, P53, GCDFP-15, vimentin, and CD21; negativity for CD3, CD10, cyclin D1, HHV8, and light chains; Ki-67 index 80%. *In situ* hybridization was positive for EBER. FISH revealed BCL6 rearrangement without BCL2 or MYC alterations. Next-generation sequencing identified variations in HLA-B, TP53, CREBBP, GNA13, SMARCA4, and BCL10.

**Figure 4:**
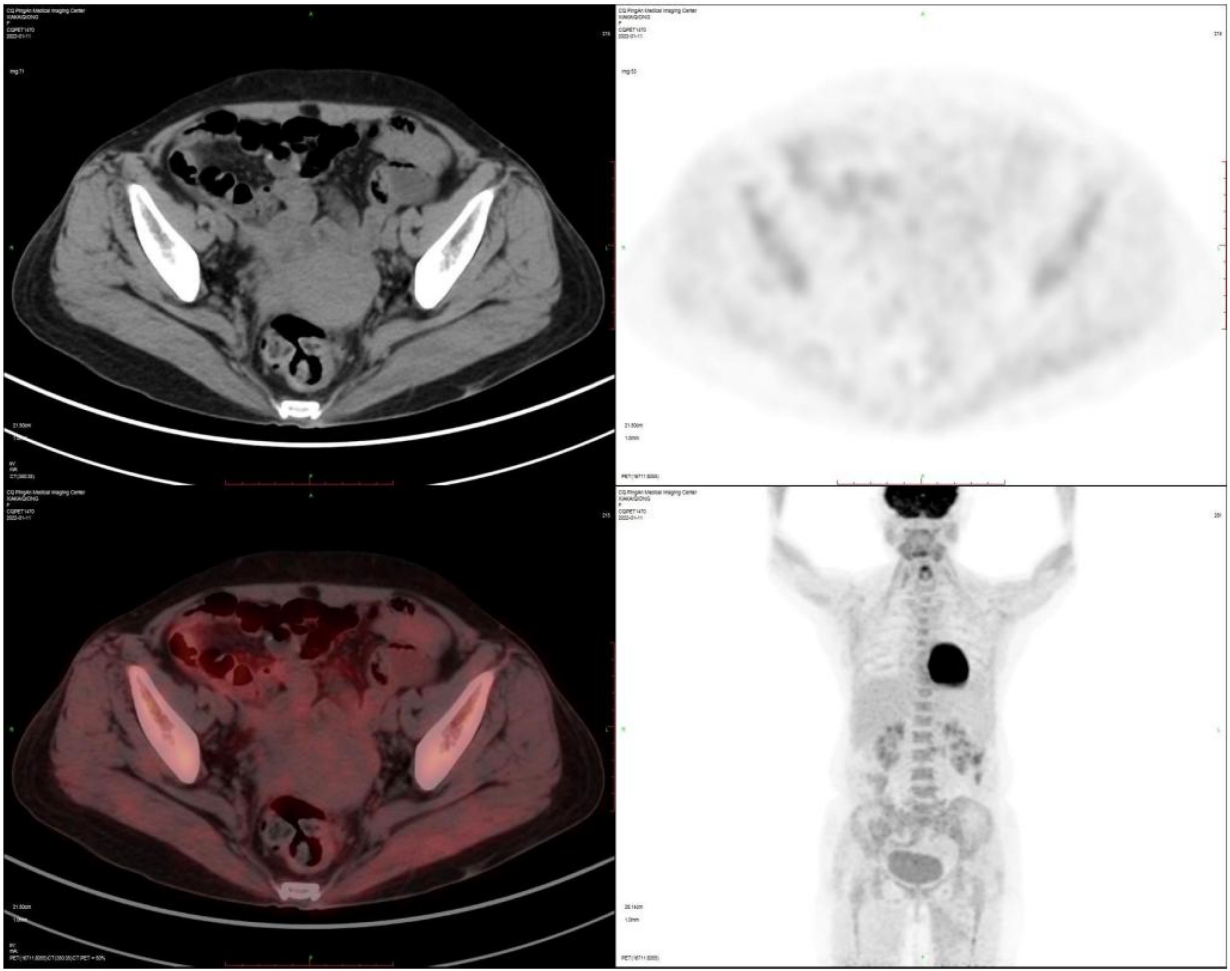
PET-CT at the end of cycle 4 chemotherapy:The lesions in the groin disappeared.

The patient was diagnosed with EBV-positive IVLBCL involving inguinal lymph nodes in the setting of AIDS. cART was switched to lamivudine/tenofovir/dolutegravir. Treatment included two cycles of CHOP followed by four cycles of R-CHOP chemotherapy starting one month after admission. Due to the presence of TP53 and BCL10 mutations, which suggest possible resistance to BTK inhibitors, and because of financial reasons, the patient refused this regimen. Notwithstanding a high-risk CNS-IPI (Central Nervous System International Prognostic Index) score, the patient provided signed refusal for CNS prophylaxis. Post-chemotherapy PET-CT showed resolution of hypermetabolism with only postoperative changes in the left groin (**Figure 4**).

After completing chemotherapy in February 2022, the patient achieved complete remission but declined stem cell transplantation. CD4+ count improved to 79 cells/μL. Treatment-related leukopenia resolved with supportive care. The patient remained in remission for two years until death due to COVID-19 in March 2023 (**Table 1**).

**Table 1:** Timeline.

2020	Diagnosed with AIDS, started 3T’C/TDF/EFV anti-HIV treatment
September 20, 2021	Recent onset of a left groin mass, fever (37.8°C), and a 5 kg weight loss. PET-CT showed disease confined to the lymph nodes, with intense hypermetabolism (Deauville score 5) in the bilateral inguinal, retroinguinal, retroperitoneal, and pelvic regions
September 30, 2021	*In situ* hybridization was positive for EBER, and FISH revealed an isolated BCL6 rearrangement. The combined pathological findings established the diagnosis of intravascular large B-cell lymphoma
October 1, 2021	The patient received CHOP/R-CHOP chemotherapyand had her antiretroviral therapy (ART)regimen changed to 3TC/TDF/DTG
January 6, 2022	After four courses of chemotherapy, the mid-term re-examination by PET-CT showed that all the lesions had disappeared. The therapeutic effect was evaluated as complete remission.
Two years after discharge	The patient remained in remission for two years until death due to COVID-19 in March 2023

## Discussion

IVLBCL is a rare large B-cell lymphoma, with an annual incidence of less than 0.5 cases per 1 million individuals. It predominantly affects the elderly, with a median age at diagnosis around 70 years, and shows no significant gender predilection (3). The disease can involve virtually any organ; central nervous system and skin are the most commonly affected sites (4), whereas lymph nodes and peripheral blood are often spared (5). The exact etiology of IVLBCL remains unclear.

When skin is involved, patients may present with painful erythematous nodules, plaques, ulcerated lesions, or macules. Neurological manifestations often mimic cerebrovascular diseases. Because of the highly variable and nonspecific clinical picture, early diagnosis is challenging, and many patients present at an advanced stage. Therefore, IVLBCL should be considered in patients with unexplained fever accompanied by neurological symptoms or cutaneous lesions. Early random deep skin biopsy can improve the diagnostic yield. The absence of classic skin manifestations in this patient, combined with the lack of random skin biopsy and thus incomplete exclusion of cutaneous involvement, represents a diagnostic limitation.

The present case concerns a 52-year-old woman whose initial symptom was a left inguinal mass, accompanied by afternoon and nocturnal fever, fatigue, anorexia, night sweats, and weight loss. Diagnosis was confirmed after complete excision of the lymph node for pathological examination. Histology revealed tumor cells proliferating exclusively within vascular spaces. These cells were positive for B-cell markers (CD20, PAX5) and negative for CD3. According to Hans’ algorithm (6), the tumor was classified as a non-GCB subtype of DLBCL (BCL-6 +, MUM-1 +, CD10 −), with a high proliferation index (Ki-67 80%). CD31 and CD34 positivity clearly highlighted tumor cells confined to the vascular lumen. Notably, IVLBCL usually occurs in HIV-negative individuals and is typically EBV-negative; however, in this case, EBER was positive in the lymph node tissue—a rare finding in IVLBCL (7).

This patient lacked typical CNS involvement, and cerebrospinal fluid studies were negative. Abdominal ultrasound showed no hepatosplenomegaly. Blood tests revealed moderate anemia. Bone marrow biopsy and smear showed no lymphomatous infiltration or hemophagocytosis. The skin was normal without rash, and PET-CT disclosed no cutaneous involvement. Consequently, the clinical presentation did not align with the conventional IVLBCL subtypes. Random skin biopsy, which has a higher diagnostic yield than bone marrow biopsy in patients with normal-appearing skin (8), was not performed; therefore, skin involvement could not be confirmed. Although tuberculosis DNA was detected in the bone marrow, and the patient had symptoms consistent with tuberculosis (fever, weight loss, night sweats), bronchoscopic lavage and lymph-node biopsy were negative for tuberculosis, and her symptoms improved without anti-tuberculosis therapy.

The pathogenesis of lymphoma is incompletely understood, but several viruses—including HIV, EBV, HHV-8, HBV, and HCV—have been implicated (9–13). In this case, the patient was co-infected with HIV, HBV, and EBV. Despite more than one year of antiretroviral therapy (ART) and anti-HBV treatment with 3TC/TDF/EFV, HIV-RNA and HBV-DNA were well controlled at admission. Nevertheless, her immune reconstitution was suboptimal (CD4^+^ count persistently low), which may have contributed to the development of IVLBCL (14).

IVLBCL follows an aggressive course with generally poor prognosis. CHOP-based chemotherapy is the standard regimen; the addition of rituximab improves outcomes in CD20-positive cases (15). Because the patient’s CD4^+^ count was below 50 cells/μL during the first two chemotherapy cycles, rituximab was initially withheld to reduce infection risk (16). An integrase inhibitor-based ART regimen was chosen to minimize drug interactions and organ toxicity (17, 18). The patient completed two cycles of CHOP followed by four cycles of R-CHOP, achieving complete remission without major opportunistic infections. During follow-up, no recurrence was observed. Unfortunately, the assessment of the disease’s natural course and treatment response was severely limited by the patient’s subsequent refusal of further therapy, the development of treatment resistance, and eventual death from an unrelated cause (COVID-19 pneumonia). Independent of these observational constraints, the presence of TP53 and BCL10 mutations, coupled with the refusal of autologous stem cell transplantation and resistance to BTK inhibitors, are themselves strong adverse prognostic factors that would likely compromise long-term survival, necessitating continued close monitoring.

In summary, IVLBCL is a rare, aggressive subtype of DLBCL with nonspecific clinical features. This case is unusual because the disease arose primarily in lymph nodes—a presentation scarcely reported. While CNS and cutaneous signs should raise suspicion for IVLBCL, unexplained lymphadenopathy should also include IVLBCL in the differential diagnosis. Pathological biopsy remains essential for definitive diagnosis and timely intervention. The mechanism underlying exclusive nodal involvement in this patient warrants further investigation. For HIV-positive patients with IVLBCL, effective ART combined with rituximab-based multi-agent chemotherapy may improve survival prospects.

## Conclusion

This case of intravascular large B-cell lymphoma (IVLBCL) demonstrates several highly atypical features: its rare occurrence as a DLBCL subtype, isolated lymph node involvement, lack of characteristic cutaneous or CNS signs, and presentation in an HIV-positive individual. Despite profound immunosuppression, complete remission was achieved through early recognition and combined antiretroviral therapy with chemotherapy. The case underscores the importance of vigilant, individualized management in such rare and diagnostically challenging scenarios.

## Data Availability

The original contributions presented in the study are included in the article/supplementary material. Further inquiries can be directed to the corresponding author.
